# Knowledge, attitudes, practices, and influencing factors of anxiety among pregnant women in Wuhan during the outbreak of COVID-19: a cross-sectional study

**DOI:** 10.1186/s12884-021-03561-7

**Published:** 2021-01-25

**Authors:** Wenping Ding, Jianmei Lu, Yan Zhou, Weizhong Wei, Zhihong Zhou, Min Chen

**Affiliations:** 1 Department of diagnostic ultrasound, Wuhan Women and Children Medical Care Center, Wuhan, 430030 China; 2 Department of Obstetrics, Wuhan Women and Children Medical Care Center, Wuhan, 430030 China; 3 Department of diagnostic ultrasound, Maternal and Children’s Hospital of Jiangxia District, Wuhan, 430100 China; 4 Department of Fetal Medicine and Prenatal Diagnosis, the Third Affiliated Hospital of Guangzhou Medical University, Guangzhou, 510150 China; 5 Obstetrics & Gynecology Institute of Guangzhou, Guangzhou, 510150 China; 6 The Medical Centre for Critical Pregnant Women in Guangzhou, Guangzhou, 510150 China

## Abstract

**Background:**

Prenatal anxiety has been a significant public health issue globally, leading to adverse health outcomes for mothers and children. The study aimed to evaluate the sociodemographic characteristics, knowledge, attitudes, and practices (KAP), and anxiety level of pregnant women during the coronavirus disease 2019 (COVID-19) epidemic in Wuhan and investigate the influencing factors for prenatal anxiety in this specific context.

**Methods:**

Pregnant subjects’ KAP towards COVID-19 and their sociodemographics and pregnancy information were collected using questionnaires. The Zung Self-Rating Anxiety Scale (SAS) was used to assess anxiety status. Factors associated with the level of prenatal anxiety were analyzed by Pearson’s chi-square test and multivariable logistic regression analyses.

**Results:**

The prenatal anxiety prevalence in this population was 20.8%. The mean score of knowledge was 13.2 ± 1.1 on a 0 ~ 14 scale. The attitudes and practices data showed that 580/ 817 (71.0%) were very concerned about the news of COVID-19, 455/817 (55.7%) considered the official media to be the most reliable information source for COVID-19, and 681/817 (83.4%) were anxious about the possibility of being infected by COVID-19. However, only 83/817 (10.2%) worried about contracting COVID-19 infection through the ultrasound transducer during a routing morphology scan. About two-thirds 528/817 (64.6%) delayed or canceled the antenatal visits. Approximately half of them 410/817 (50.2%) used two kinds of personal protection equipments (PPEs) during hospital visits. Logistic regression analysis revealed that the influential factors for prenatal anxiety included previous children in the family, knowledge score, media trust, worry of contracting the COVID-19 infection and worry about getting infected with COVID-19 from the ultrasound probe antenatal care (ANC) schedule.

**Conclusion:**

Prenatal anxiety was prevalent among pregnant women in Wuhan during the outbreak of COVID-19. The current findings identified factors associated with the level of prenatal anxiety that could be targeted for psychological care.

**Supplementary Information:**

The online version contains supplementary material available at 10.1186/s12884-021-03561-7.

## Background

According to the Chinese Center for Disease Control and Prevention (China CDC), since the identification of the first case of COVID-19 in Wuhan on 8 December 2019, a total of 82,874 confirmed cases of COVID-19 and 4633 deaths due to the disease were recorded at the end of April in China [1]. The disease has then spread to Southeast Asia, Europe, North America, Australia, and the Middle East, leading to the COVID-19 pandemic declared by the World Health Organization (WHO) on 11 March 2020 [2].

COVID-19 is caused by SARS-CoV-2, a new human-infecting betacoronavirus different from SARS-CoV-1 and MERS-CoV [3]. It is spread through respiratory droplets and direct contact [4]. There are only a few cases of pregnant women with COVID-19. It remains unclear whether intrauterine infection can be caused by vertical transmission in women who contract COVID-19 during pregnancy [5–7]. Limited data suggest that pregnant women with a confirmed COVID-19 infection were similar to their non-pregnant counterparts in the general population with relatively optimistic clinical outcomes [8, 9]. A meta-analysis suggested that pregnant and recently pregnant women are less likely to manifest covid-19 related symptoms of fever and myalgia than non-pregnant women of reproductive age and are potentially more likely to need intensive care treatment for COVID-19 [10]. However, most research has mainly focused on the therapeutic aspects, while pregnant women’s mental health status during the COVID-19 is much less studied.

Prenatal care is vital to a healthy pregnancy [11]. Any non-routine changes to prenatal care could be a stress factor to pregnant women, especially in Wuhan, the first city hit by the virus. Since the outbreak, the Wuhan government has taken several unprecedented precautionary measures. All the suspected or confirmed COVID-19 case with pregnancy were not allowed to visit the maternal hospitals, only designated hospitals. As for maternal hospitals, all prenatal exercise, hospital tours, and prenatal classes were canceled. Mask wearing became mandatory in hospitals. Only routing obstetric and gynecological scans were not called off, and the preparation and cleaning of ultrasound equipment and transducer was in accordance with local guidelines [12]. Antenatal care was available, but the delivery of it was affected. The pregnant women were recommended to reduce the antenatal visit.

Prenatal anxiety has important maternal-fetal implications. It may be associated with preterm birth, fetal growth restriction, and obstetric complications and had enduring effects directly or indirectly on children’s growth and development [13]. Children with a history of in utero exposure to maternal anxiety are at increased risk for various neuropsychiatric conditions such as attention-deficit/hyperactivity disorder (ADHD) [14, 15]. The anxiety level is affected by individual personality, resilience, education level, support from family, satisfaction with life quality, occupation, and financial status [16, 17]. During this pandemic, anxiety may also be affected by individuals’ knowledge, attitudes, and practices (KAP) towards COVID-19 [18, 19]. For example, increased anxiety levels have been reported in countries and cities in the general population and subpopulation with significant outbreaks [19–21]. But only a few studies investigate the anxiety level and its influencing factors among pregnant women [22–24]. The study aimed to evaluate the sociodemographic characteristics, KAP, and anxiety level of pregnant women during the COVID-19 epidemic in Wuhan and investigate the affecting factors for prenatal anxiety in this specific context.

## Methods

### Participants

This cross-sectional survey was conducted from March 7–23, 2020, in Wuhan. All data were collected online. An informational leaflet was sent to each woman waiting for a routine transabdominal obstetric ultrasound examination in two maternal hospitals in the Jiangun (urban) and Jianxia (suburban) districts, which are similar in practice. It included a brief introduction to the study, notes for filling out the questionnaire, and the online survey’s QR code. A survey tool, “wen juan xing,” a product made in China, https://www.wjx.cn/. was used. Inclusion criteria were gestational weeks around 10th to 40th week; no personal history and family history of mental disorders; no previous history of severe somatic diseases; ability to understand the questionnaire’s content and complete it independently; and willingness to participate in the survey. Exclusion Criteria were somatic diseases, fetal abnormalities, those who cannot understand the questionnaire’s contents (due to mental retardation or low cultural level). A pilot study was performed on 25 participants, and their feedback was used to modify and improve the questionnaire. The Ethics Committee of the Wuhan Women and Children Medical Care Center approved this study. (see Supplementary files 1 for details).

### Data collection

The questionnaire contained three parts; sociodemographic characteristics, KAP, and Zung self-rating anxiety scale (SAS); all of them were prepared in Chinese. (see Supplementary files 2 for details).

Sociodemographic characteristics included maternal age, gestational age, occupation, educational level, household income, previous children in the family, reproductive history, and complications during the pregnancy.

Participants’ knowledge of COVID-19 was assessed using six questions: 1) What is the cause of COVID-19; 2) Which population is susceptible; 3) Is there an effective treatment for COVID-19; 4) What are the routes of transmission for COVID-19; 5) What are the main clinical symptoms of COVID-19; and 6) How can the public prevent COVID-19. The first three questions had a single answer, while the last three questions had multiple choices. Each correct answer was coded one. Both incorrect responses and “I don’t know.” were coded zero. The scoring range of the questionnaire was 0 to 14. Knowledge scores for individuals were summed to a total score. (see Table 2 for details).

Attitudes toward COVID-19 was assessed using four single choice questions: 1) What was the level of attention to the news of COVID-19?; 2) What media do you trust?; 3) How much did you worry about the contraction of the COVID-19 infection?; and 4) Were you worried about getting infected with COVID-19 by the ultrasound probe? The practice was assessed using three questions: 1) How did you schedule antenatal care (ANC) during the outbreak of COVID-19?; 2) How many kinds of PPEs were used when you were in the hospital for the obstetric ultrasound examinations?; 3) Did you put on the gown in the hospital? (see Table 3 for details).

### Anxiety assessment criteria

The anxiety of pregnant women was measured with the 20-item self-rating anxiety scale (SAS), developed by Zung in 1971 [25]. A Chinese version of the SAS was used to assess the participants’ general anxiety on a 4-point scale from 1 (none or little of the time) to 4 (most or all). Though many contemporary instruments have been validated for use in pregnant, this Chinese version of the SAS has been used in the Chinese population with popularity and demonstrated satisfactory reliability and validity. The anxiety domains and psychometric properties have been established for use in Chinese population [26–29].

Higher scores suggest a higher degree of anxiety. Standardization was performed based on the SAS (raw data multiplied by 1.25). A score of more than 50 is considered to be anxiety, which is then classified as mild anxiety (50–59), moderate anxiety (60–69), and severe anxiety (≥ 70). In this study, we only explored whether pregnant women have anxiety symptoms.

### Statistical analysis

Statistical analyses were performed with the Statistical Analysis System, version 9.4 (Cary, North Carolina). Continuous variables were presented as mean ± SD or median with interquartile range (IQR), while categorical variables were presented as absolute frequency and percentages. Pearson’s chi-square test was used to examine associations between prenatal stress and categorical risk factors. Univariate and multivariate logistic regression analyses were used to quantify the associations between risk factors and prenatal anxiety. A stepwise procedure was used to select the final model. Two-sided *P*-values < 0.05 were considered statistically significant.

## Results

### Participants’ sociodemographic characteristics

One thousand and eighty-five women were approached; 268 of them declined or unable to complete any or part of the questionnaire. A total of 817 pregnant women were recruited. The mean maternal age was 29.1 ± 4.0 years. Table 1 presents the sociodemographic characteristics and anxiety data, in which 94.6% (773/817) of all participants completed at least a senior high school education. More than one-third of participants (335/817, 41.0%) were company employees, nearly one-quarter (185/817, 22.6%) were unemployed, and the rest had different kinds of occupations, including civil servants (137/817, 16.8%), self-employed (73/817, 8.9%), farmers (13/817, 1.6%), and others. Most participants were in their third trimester (455/817, 55.7%),

**Table 1:** Sociodemographic characteristics and univariate analysis

Variables	Total N (%)	Non-anxiety N (%)	Anxiety N (%)	P
**Age (years)**				
< 20	3 (0.3)	2 (66.7)	1 (33.3)	0.126
20–25	88 (10.8)	69 (78.4)	19 (21.6)	
25–30	345 (42.2)	281 (81.4)	64 (18.6)	
30–35	292 (35.7)	233 (79.8)	59 (20.2)	
35–40	78 (9.5)	55 (70.5)	23 (29.5)	
> 40	11 (1.3)	7 (63.6)	4 (36.4)	
**Trimester**				
First	115 (14.1)	91 (79.1)	24 (20.9)	0.952
Second	247 (30.2)	195 (78.9)	52 (21.1)	
Third	455 (55.7)	361 (79.3)	94 (20.7)	
**Occupation**				
Civil servant	137 (16.8)	107 (78.1)	30 (21.9)	0.474
Company staff	335 (40.0)	273 (81.4)	62 (18.6)	
Self-employed	73 (8.9)	54 (74.0)	19 (26.0)	
Farmer	13 (1.6)	12 (92.3)	1 (7.7)	
Housewife	185 (22.6)	145 (78.4)	40 (21.6)	
Student	2 (0.2)	1 (50.0)	1 (50.0)	
others	73 (8.9)	56 (76.7)	17 (23.3)	
**Household income**				
< 4000	146 (17.9)	114 (78.1)	32 (21.9)	0.486
4000–6000	279 (34.1)	216 (77.4)	63 (22.6)	
6000–10,000	239 (29.3)	196 (82.0)	43 (18.0)	
> 10,000	153 (18.7)	121 (79.1)	32 (20.9)	
**Education**				
Junior high school and below	44 (5.4)	29 (65.9)	15 (34.1)	0.026
Senior high school and above	773 (94.6)	618 (79.9)	155 (20.1)	
**Reproductive history**				
Naturally-conceived	747 (91.4)	595 (79.7)	152 (20.3)	0.290
Non-naturally-conceived	70 (8.6)	52 (74.3)	18 (25.7)	
Previous children in the family				
No	565 (61.2)	460 (81.4)	105 (18.6)	0.019
Yes	252 (30.8)	187 (74.2)	65 (25.8)	
**Complications**				
No	710 (86.9)	569 (80.1)	141 (19.9)	0.085
Yes	107 (13.1)	78 (72.9)	29 (27.1)	

while 14.1% (115/817) and 30.2% (247/817) were in their first trimester and second trimester, respectively. Meanwhile, 13.1% (107/817) had obstetric complications, and 91.4% (747/817) conceived naturally.

### The prevalence of prenatal anxiety during pregnancy

One hundred and seventy out of 817 (170/817, 20.8%) pregnant women had anxiety with a SAS score of ≥50. One hundred and fifteen women were enrolled in this cohort study during their first trimester, two hundred and forty-seven in their second, and four hundred and fifty-five in their third trimester. The prevalence of anxiety was 20.9, 21.1, and 20.7% in the first, second, and third trimesters, respectively (see Table 1).

### KAP on COVID-19

The mean knowledge score was 13.2 ± 1.1. The current study demonstrated that only 55.8% (456/817) knew that no effective treatment for COVID-19 was available. Nearly one-fifth of the participants (161/817, 19.7%) did not know that the general population is susceptible to infection. However, almost all (752/817, 92.0%) knew that a novel coronavirus causes COVID-19. Furthermore, nearly all of them knew the main clinical presentations of COVID-19 and how to protect themselves (see Table 2 for details).

**Table 2:** Questions of knowledge towards COVID-19

Questions	Answer (% of the total sample)		
	True	False	I don’t know
The whole population is susceptible to COVID-19	656 (80.3%)	98 (11.2%)	63 (7.7%)
The COVID-19 is caused by coronavirus	752 (91.9%)	14 (1.7%)	51 (6.2%)
There is no efficient treatment for COVID-19	456 (55.7%)	226 (27.7%)	135 (16.5%)
What are the routes of transmission for COVID-19			
1. Respiratory droplets	812 (99.4%)	1 (0.0%)	6 (0.1%)
2. Close contacts	805 (98.6%)	7 (0.1%)	9 (1.3%)
The main clinical symptoms of COVID-19			
1. Fever	813 (99.5%)	0 (0.0%)	4 (0.0%)
2. Fatigue	804 (98.1%)	1 (0.0%)	13 (1.8%)
3. Dry cough	802 (98.4%)	3 (0.0%)	13 (1.8%)
How can the public prevent COVID-19			
1. Wear a mask when going out	816 (99.9%)	0 (0.0%)	1 (0.0%)
2. Wash your hands frequently	816 (99.9%)	0 (0.0%)	1 (0.0%)
3. Avoid public places	816 (99.9%)	0 (0.0%)	1 (0.0%)
4. Open the window frequently for ventilation	814 (99.6%)	2 (0.0%)	1 (0.0%)
5. Balance work and rest	803 (98.2%)	8 (1.0%)	6 (0.1%)
6. Reasonable diet	780 (95.6%)	20 (2.4%)	17 (2.1%)

More than half (456/817, 55.7%) of the participants considered the official media to be the most reliable source of information towards COVID-19. The majority (681/817, 83.4%) were anxious about being infected by COVID-19. However, about one-tenth (83/817, 10.2%) of the participants remained worried about contracting COVID-19 infection by the transducer.

About two-thirds (528/817, 64.6%) of the participants delayed or canceled the antenatal visits and prenatal ultrasound examinations. When they were asked about the use of PPE, approximately one-fifth of them (148/817, 18.1%) wore only one kind of PPE except a face mask, one-quarter (211/817, 25.8%) wore a protective gown or suit.

### The influencing factors of prenatal anxiety

Regression analysis showed the level of prenatal anxiety were associated with previous children in the family, education, knowledge towards COVID-19, trust in the media, worry about contracting the COVID-19 infection, and worry about getting the COVID-19 infection from the ultrasound transducer (*p* < 0.05; Tables 1, 3) In the multivariable model of sociodemographic characteristics (Table 4), previous children in the family increased the odds of prenatal anxiety (*OR* = 1.600, 95% CI: 1.104, 2.319). No significant differences were found in education between Junior high school and below senior high school and above.

**Table 3:** Attitudes and practice characteristics of participants and univariate analysis

Variables	Total N (%)	Non-anxiety N (%)	Anxiety (N, %)	P
**Attention to the news of COVID-19**				
Very concern	580 (71.0)	453 (78.1)	127 (21.9)	0.199
Concern	208 (25.5)	173 (83.2)	35 (16.8)	
Not every concern	29 (3.5)	21 (72.4)	8 (27.6)	
**Media trust**				
Non official	362 (44.3)	270 (74.6)	92 (25.4)	0.004
Official	455 (55.7)	377 (82.9)	78 (17.1)	
**Worried about contracting COVID-19**				
Very worried	681 (83.4)	522 (76.7)	159 (23.3)	< 0.001
Somewhat worried or not worried	136 (16.6)	125 (91.9)	11 (8.1)	
**Worried about contracting COVID-19 by the probe**^a^				
Yes	83 (10.2)	55 (6.3)	28 (33.7)	0.002
No or don’t know	734 (89.8)	592 (80.7)	142 (19.3)	
**ANC schedule**				
Postpone or reduce times	528 (64.6)	431 (81.6)	97 (18.4)	0.028
other	289 (35.4)	204 (70.6)	85 (29.4)	
**Kind of PPE used in the hospital (except facemask)**				
One	148 (18.1)	111 (75.0)	37 (25.0)	0.448
Two	410 (50.2)	331 (80.7)	79 (19.3)	
Three or more	259 (31.7)	205 (79.2)	54 (20.8)	
**With gown in the hospital**				
Yes	211 (25.8)	163 (77.3)	48 (22.7)	0.420
No	606 (74.2)	484 (79.9)	122 (20.1)	

^a^The cleaning of ultrasound transducer was in accordance with local guidelines

**Table 4:** Univariate and multivariate logistic regression analyses of influencing factors of anxiety

Variables	Univariable		Multivariable	
	OR(95%CI)	*P*-value	aOR(95%CI)^a^	*P*-value
**Knowledge scores**	0.828 (0.719–0.953)	0.009	0.847 (0.724–0.990)	0.037
**Previous children in the family**				
No	Ref		Ref	
Yes	1.525 (1.068–2.178)	0.020	1.600 (1.104–2.319)	0.013
**Education**				
Junior high school and below	Ref		Ref	
Senior high school and above	0.485 (0.254–0.927)	0.029	0.912 (0.430–1.932)	0.809
**Media trust**				
Non-official	Ref		Ref	
Official	0.607 (0.432–0.853)	0.004	0.620 (0.434–0.885)	0.008
**Worried about contracting COVID-19**				
Very worried	Ref		Ref	
Somewhat worried or not worried	0.289 (0.152–0.549)	< 0.001	0.310 (0.161–0.594)	< 0.001
**Concerned about contracting COVID-19 by the probe**				
Yes	Ref		Ref	
No	0.471 (0.289–0.769)	0.003	0.514 (0.308–0.857)	0.011
**ANC schedule**				
Postponed ANC or reduce the times	Ref		Ref	
Not postpone ANC or reduce the times	1.481 (1.042–2.106)	0.029	1.446 (1.003–2.086)	0.048

^a^ Data are multivariable-adjusted OR

**Fig. 1:**
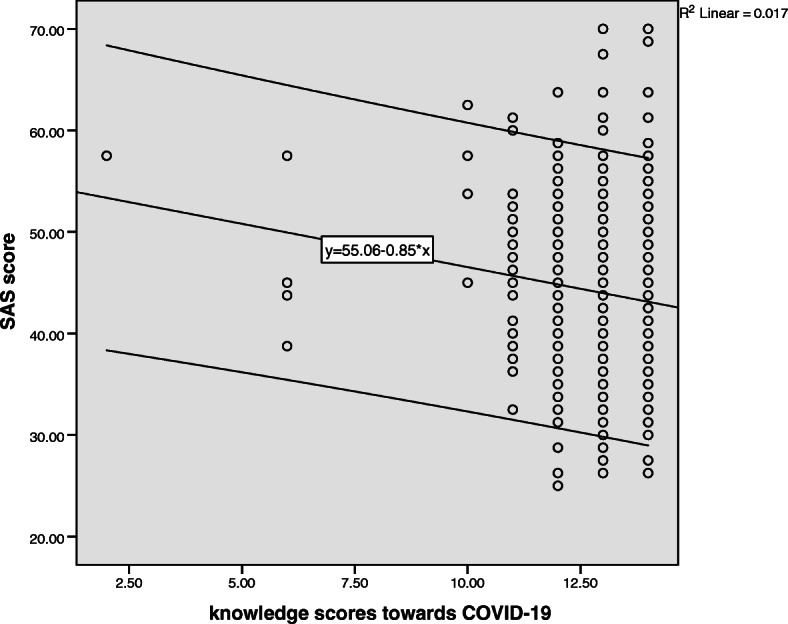
The association between SAS score and knowledge score

In terms of KAP factors, women with higher knowledge scores were less likely to have anxiety symptoms than those with lower scores (*OR* = 0.847, 95% CI: 0.724, 0.990; Table 4, Fig. 1). And women who trusted in official media were less likely to have anxiety symptoms than those who did not trust official media (*OR* = 0.620, 95% CI: 0.434, 0.885). Similar to women who did not worry significantly about contracting the COVID-19 infection (*OR* = 0.310, 95% CI: 0.161, 0.594), women who did not worry about getting the COVID-19 infection from the ultrasound transducer had lower odds of prenatal anxiety as well (*OR* = 0.514, 95% CI: 0.308, 0.857). Moreover, women who did not postpone their antenatal appointments had a higher risk of anxiety than those who did (*OR* = 1.446, 95% CI: 1.003, 2.086).

## Discussion

The current study showed that pregnant women in Wuhan had good knowledge, attitudes, practices towards COVID-19. Most women paid close attention to the official news on COVID-19 and did not worry about being infected by the transducer. Almost two-thirds of the participants either postponed their antenatal visits or reduced the visits’ frequency as advised by the government. Still, about 10.2% of pregnant women were worried about contracting COVID-19 by the transducer and had high anxiety levels. It is suggested that doctors and nurses should pay more attention to this issue.

The current findings revealed the highest prevalence of anxiety (21.1%) in participants who were in the second trimester, the lowest in those who were in the third trimester (20.7%), and a middle level of anxiety was in those who were in the first trimester (20.9%). These differences are tiny. The general prevalence of anxiety was 20.8%, similar to that in a global study (18.2–24.6%) before the COVID-19 pandemic [30]. However, It was a little different from the previous study that reported that women close to the term are more worried than those who aren’t [30]. Compared with the other cities in China before the COVID-19 pandemic, the prevalence of anxiety in the current study was higher than in Chongqing (15.0%) but lower than in Zhoushan (22.6%) [17, 31]. And literature reported the prevalence of anxiety in Wuhan (24.5%) was much higher than in Chongqin (10.4%) during the pandemic [24]. Meanwhile, Wu and his colleges reported a clinically significant increase in the prevalence of depressive and anxiety symptoms after the declaration of human-to-human transmission [32]. During the remission phase of COVID-19 in southern China, the anxiety rate was 31.2% [33]. Hence, the anxiety level among pregnant women during the outbreak of COVID-19 in Wuhan in our study was intermediate in China generally. It may not be affected a lot by the pandemic and trimester. This was likely due to increased available information and reassurance through social media, healthcare professionals, and primary care. Moreover, prenatal anxiety levels were different from studies, attributed to the study designs [34, 35].

As noted in the statistical modeling, having previous children in the family was the only risk factor among sociodemographic characteristics for prenatal anxiety. It’s consistent with previous studies. The prevalence of anxiety in women pregnant with their second child was relatively high in China [26]. It’s because they were worried about her child and older relatives being infected by Covid-19, leading to an increase in prenatal anxiety, and physical activity changes resulting from pregnancy were significantly correlated with anxiety disorder [36]. However, their fear was not affected by advanced maternal age, complications during the pregnancy, or household income. The reason might be that most of the participants had learned antenatal care knowledge during their last pregnancy. And 94.6% of pregnant women had an senior high school education or above, they were easier to access the correct information. Moreover, the universal coverage of maternity insurance in China has removed concerns about medical expenses [37].

The current study assessed KAP towards COVID-19 that could impact prenatal anxiety. The followings were protective factors for prenatal anxiety, including more knowledge of COVID-19, not worrying much about contracting the virus, not worrying about getting infected by the probe, trusting in official media, and postponed or reduced ANC times. These findings have implied clinical and policy implications as the COVID-19 epidemic continue to spread. Firstly, health administrators should provide accurate and updated information continuously. Secondly, this highlighted the care of pregnant women should be tailored individually for women’s mental health [38].

### Limitations

Our study has several limitations. Firstly, the current study was conducted in a single period during the COVID-19 outbreak. A random selection from the general population was not available. Secondly, all participants were recruited from maternal hospitals, which may introduce selection bias. Thirdly, the study did not have a control group (non-Covid-19-time control group) because it was not possible at the survey time.

Moreover, all the data in this study were collected through the online questionnaire, which we did not evaluate its reliability and validity. However, the current study used standardized scales to assess the anxiety symptom. Finally, self-reported levels of anxiety may not always be aligned with assessments by mental health professionals.

## Conclusion

During the intermediate phase of the COVID-19 outbreak in Wuhan, pregnant women had an overall good knowledge of COVID-19, and anxiety was common. We have identified several influencing factors of prenatal anxiety, which can guide public health strategy regarding pregnant women anxieties.

## Supplementary Information

**Additional file 1.** The proof of license. It’s a PDF copy of the license from the Ethics Committee of the Wuhan Women and Children Medical Care Center.

**Additional file 2.** The full English language version of the questionnaire. The full English language version of the questionnaire contained all the details of the original Chinese version of the questionnaire.

## Data Availability

The data sets generated and analyzed during the current study are not publicly available due to identifiable information but are available from the corresponding author on reasonable request. answering the survey.

## Abbreviations

KAP: Knowledge, attitudes, and practices
COVID-19: The coronavirus disease 2019;
SAS: Zung self-rating anxiety scale
ANC: Antenatal care
WHO: World Health Organization
CDC: Center for Disease Control and Prevention
ADHD: Attention-deficit/hyperactivity disorder
PPE: Personal protection equipment
SD: Standard deviation
IQR: Interquartile range
OR: Odds ratio
