# Supplementary material for: Knowledge, attitudes, practices, and influencing factors of anxiety among pregnant women in Wuhan during the outbreak of COVID-19: a cross-sectional study
Source: BMC Pregnancy Childbirth. 2021 Jan 25;21:80. doi: 10.1186/s12884-021-03561-7 (PMC7829651; doi:10.1186/s12884-021-03561-7)
Supplement: Supplementary file 1 — Additional file 1. The proof of license. It’s a PDF copy of the license from the Ethics Committee of the Wuhan Women and Children Medical Care Center. [file 12884_2021_3561_MOESM1_ESM.zip › licence2R2.pdf]

附件：

审查文件清单　　伦理审查编号：2021R003　　审查日期：2021/01/04

编号	名称	版本号	版本日期
1	初审申请表	/	/
2	研究方案	1.0	2020/03/15
3	知情同意书	1.0	2020/03/15
4	主要研究者简历及研究小组成员名单	/	/
