# Supplementary material for: Knowledge, attitudes, practices, and influencing factors of anxiety among pregnant women in Wuhan during the outbreak of COVID-19: a cross-sectional study
Source: BMC Pregnancy Childbirth. 2021 Jan 25;21:80. doi: 10.1186/s12884-021-03561-7 (PMC7829651; doi:10.1186/s12884-021-03561-7)
Supplement: Supplementary file 1 — Additional file 1. The proof of license. It’s a PDF copy of the license from the Ethics Committee of the Wuhan Women and Children Medical Care Center. [file 12884_2021_3561_MOESM1_ESM.zip › license1R2.pdf]

伦理审查批件

伦理审查编号：2021R003　　　　审查日期：2021/01/04

项目名称	新冠期间孕妇知识行为调查及焦虑相关因素分析		
药物名称	/		
申办者	/		
CRO	/		
研究单位/专业	妇幼超声科	主要研究者	丁文萍
审查地点	武汉市香港路 100 号 武汉儿童医院医学伦理委员会		
审查类别	初始审查	审查方式	快速审查
审查委员	郭怡阳，胡红兵		
审查文件	可添加附件		
审查意见	1. 经本伦理委员会审查，同意该项目按批准的试验方案开展临床试验；<br>2. 本批件有效期为一年，自 2020 年 12 月 31 日至 2021 年 12 月 30 日。<br><br>注意（请仔细阅读）：<br>1. 临床研究应遵循中国《涉及人的生物医学研究伦理审查办法》、《药物临床试验质量管理规范》、《药物临床试验伦理审查工作指导原则》、《赫尔辛基宣言》和 ICH-GCP 及相关法律法规。<br>2. 本临床试验应在批准之日起一年内实施，逾期未实施的，本批件自行废止。<br>3. 涉及人类遗传资源采集、收集、买卖、出口、出境的研究项目，须在获得人类遗传资源办公室的批件后开展。<br>4. 研究过程中若变更主要研究者，对临床研究方案、知情同意书、招募材料等任何修改，请提交修正案审查申请。<br>5. 研究过程中发生严重不良事件，请及时递交严重不良事件报告，当出现任何可能显著影响试验进行、或增加受试者危险的情况时，请及时向伦理委员会提出书面报告。<br>6. 纳入不符合纳入标准的受试者；研究过程中，符合提前终止的研究标准而没有让受试者退出；给予受试者错误的治疗或不正确的剂量；给予受试者方案禁用的合并用药等没有遵从方案开展研究的情况，请及时递交违背方案报告。<br>7. 暂停或提前终止临床试验，需及时提交暂停/终止研究报告。<br>8. 根据伦理委员会对跟踪审查频率的意见，请在批件失效前 1 个月递交持续审查申请，期间伦理委员会有权根据研究进展情况改变跟踪审查频度。<br>9. 研究结束时，须向伦理委员会提交结题报告。		
出席委员	/		
投票结果	/		

主任委员（签字）　　　　日期：2021 年 01 月 04 日

武汉儿童医院医学伦理委员会（盖章）

（印章：武汉儿童医院 武汉市妇幼保健院 … 伦理委员会）
