# Supplementary material for: Knowledge, attitudes, practices, and influencing factors of anxiety among pregnant women in Wuhan during the outbreak of COVID-19: a cross-sectional study
Source: BMC Pregnancy Childbirth. 2021 Jan 25;21:80. doi: 10.1186/s12884-021-03561-7 (PMC7829651; doi:10.1186/s12884-021-03561-7)
Supplement: Supplementary file 2 — Additional file 2. The full English language version of the questionnaire. The full English language version of the questionnaire contained all the details of the original Chinese version of the questionnaire. [file 12884_2021_3561_MOESM2_ESM.docx]

**The main content of the questionnaire**

**Part I ：Sociodemographic characteristics**

**Age** [ single choice ]

| ○ ≤20Ys |
| --- |
| ○ 20-25Ys |
| ○ 25-30Ys |
| ○ 30-35Ys |
| ○ 35-40Ys |
| ○ ≥40Y |

**Gestational age:** [single choice]

| ○ first trimester（≤13^+6^ weeks） |
| --- |
| ○ second trimester（13^+6^—28 weeks） |
| ○ third trimester（≥28weeks） |

**Occupation:** [single choice]

| ○ Civil servant |
| --- |
| ○ Company staff |
| ○ Self-employed |
| ○ Farmer |
| ○ Housewife |
| ○ Student |
| ○ Others |

**Pregnancy complication:** [single choice]

| ○Yes：  ①gestational diabetes mellitus；②hypertensive disorder；③placenta previa；  ④preterm birth ⑤fetal- growth disorders |
| --- |
| ○ No |

**Reproductive history** [single choice]

| ○ Naturally-conceived |
| --- |
| ○ Non-naturally-conceived |

**Previous children in the family** [single choice]

| ○ Yes |
| --- |
| ○ No |

**Education:** [single choice]

| ○ Junior high school and below |
| --- |
| ○ Senior high school and above |

**Household income** [single choice]

| ○ ≤4000 |
| --- |
| ○ 4000-6000 |
| ○ 6000-10000 |
| ○ ≥10000 |

**Part II ：Knowledge，attitudes and practices towards COVID-19**

**The whole population is susceptible to COVID-19.** [single choice]

| ○ True |
| --- |
| ○ False |
| ○ I do not know. |

**A coronavirus causes the COVID-19.** [single choice]

| ○ True |
| --- |
| ○ False |
| ○ I do not know. |

**There is no efficient treatment for COVID-19.** [single choice]

| ○ True |
| --- |
| ○ False |
| ○ I do not know. |

**What are the routes of transmission for COVID-19？** [single matrix choice]

|  | True | False | I do not know. |
| --- | --- | --- | --- |
| 1. Respiratory droplets | □ | □ | □ |
| 2. Close contacts | □ | □ | □ |

**The main clinical symptoms of COVID-19**: [single matrix choice]

|  | True | False | I do not know. |
| --- | --- | --- | --- |
| 1. Fever | □ | □ | □ |
| 2. Fatigue | □ | □ | □ |
| 3. Dry cough | □ | □ | □ |

**How can the public prevent COVID-19?**[single matrix choice]

|  | True | False | I do not know. |
| --- | --- | --- | --- |
| 1. Wear a mask when going out | ○ | ○ | ○ |
| 2. Wash your hands frequently | ○ | ○ | ○ |
| 3. Avoid public places | ○ | ○ | ○ |
| 4. Open the window frequently for ventilation | ○ | ○ | ○ |
| 5. Balance work and rest | ○ | ○ | ○ |
| 6. Reasonable diet | ○ | ○ | ○ |

**Which media do you trust?** [single choice]

| ○ Non-official |
| --- |
| ○ Official |

**Attention to the news of COVID-19** [single choice]

| ○ Very concern |
| --- |
| ○ Concern |
| ○ Not concern |

**How are you worried about contracting COVID-19 during the outbreak?** [single choice]

| ○ Very worried |
| --- |
| ○ Somewhat worried or not worried |

**Do you concerned about contracting COVID-19 by the probe?** [single choice]

| ○ Yes |
| --- |
| ○ No |

**How do you schedule your antenatal care?** [single choice]

| ○ Postpone or reduce visit |
| --- |
| ○ Others |
|  |

**Which kind of PPE do you put on in the hospital?** [multiple choice]

| □ Cap |
| --- |
| □ Face shield or glasses |
| □ Gloves of hands |
| □ Gloves of foot |

**Do you put on the gown in the hospital?**

| □ Yes |
| --- |
| □ No |

Zung Self-Rating Anxiety Scale (SAS)

For each item below, please place a checkmark in the column which best describes how often you felt or behaved this way during the past several days.

|  | A little of the time | Some of the time | A good part of the time | Most of the time |
| --- | --- | --- | --- | --- |
| 1.I feel more nervous and anxious than usual. | ○ | ○ | ○ | ○ |
| 2.I feel afraid for no reason at all. | ○ | ○ | ○ | ○ |
| 3.I get upset easily or feel panicky. | ○ | ○ | ○ | ○ |
| 4.I feel like I'm falling apart and going to pieces. | ○ | ○ | ○ | ○ |
| 5.I feel that everything is all right and nothing wrong will happen. | ○ | ○ | ○ | ○ |
| 6.My arms and legs shake and tremble. | ○ | ○ | ○ | ○ |
| 7.I am bothered by headaches, neck, and back pain. | ○ | ○ | ○ | ○ |
| 8.I feel weak and get tired quickly. | ○ | ○ | ○ | ○ |
| 9.I feel calm and can sit still easily. | ○ | ○ | ○ | ○ |
| 10.I can feel my heart beating fast. | ○ | ○ | ○ | ○ |
| 11.I am bothered by dizzy spells. | ○ | ○ | ○ | ○ |
| 12.I have fainting spells or feel like it. | ○ | ○ | ○ | ○ |
| 13.I can breathe in and out quickly. | ○ | ○ | ○ | ○ |
| 14.I get feelings of numbness and tingling in my fingers & toes. | ○ | ○ | ○ | ○ |
| 15.I am bothered by stomach aches or indigestion. | ○ | ○ | ○ | ○ |
| 16.I have to empty my bladder often. | ○ | ○ | ○ | ○ |
| 17.My hands are usually dry and warm. | ○ | ○ | ○ | ○ |
| 18.My face gets hot and blushes. | ○ | ○ | ○ | ○ |
| 19.I fall asleep quickly and get a good night's rest. | ○ | ○ | ○ | ○ |
| 20.I have nightmares. | ○ | ○ | ○ | ○ |
